# Interaction of the *Escherichia coli* HU Protein with Various Topological Forms of DNA

**DOI:** 10.3390/biom11111724

**Published:** 2021-11-19

**Authors:** Li Huang, Zhenfeng Zhang, Roger McMacken

**Affiliations:** 1 Department of Biochemistry and Molecular Biology, Bloomberg School of Public Health, Johns Hopkins University, Baltimore, MD 21205, USA; rmcmacke@phnet.sph.jhu.edu; 2 State Key Laboratory of Microbial Resources, Institute of Microbiology, Chinese Academy of Sciences, Beijing 100101, China

**Keywords:** HU, DNA supercoiling, affinity, chemical crosslinking

## Abstract

*E. coli* histone-like protein HU has been shown to interact with different topological forms of DNA. Using radiolabeled HU, we examine the effects of DNA supercoiling on HU–DNA interactions. We show that HU binds preferentially to negatively supercoiled DNA and that the affinity of HU for DNA increases with increases in the negative superhelical density of DNA. Binding of HU to DNA is most sensitively influenced by DNA supercoiling within a narrow but physiologically relevant range of superhelicity (*σ* = −0.06–0). Under stoichiometric binding conditions, the affinity of HU for negatively supercoiled DNA (*σ* = −0.06) is more than 10 times higher than that for relaxed DNA at physiologically relevant HU/DNA mass ratios (e.g., 1:10). This binding preference, however, becomes negligible at HU/DNA mass ratios higher than 1:2. At saturation, HU binds both negatively supercoiled and relaxed DNA with similar stoichiometries, i.e., 5–6 base pairs per HU dimer. In our chemical crosslinking studies, we demonstrate that HU molecules bound to negatively supercoiled DNA are more readily crosslinked than those bound to linear DNA. At in vivo HU/DNA ratios, HU appears to exist predominantly in a tetrameric form on negatively supercoiled DNA and in a dimeric form on linear DNA. Using a DNA ligase-mediated nick closure assay, we show that approximately 20 HU dimers are required to constrain one negative supercoil on relaxed DNA. Although fewer HU dimers may be needed to constrain one negative supercoil on negatively supercoiled DNA, our results and estimates of the cellular level of HU argue against a major role for HU in constraining supercoils in vivo. We discuss our data within the context of the dynamic distribution of the HU protein in cells, where temporal and local changes of DNA supercoiling are known to take place.

## 1. Introduction

Bacterial chromosome is organized into a highly compact and ordered structure by nucleoid-associated proteins (NAPs), and the architecture of the structure is also modulated by DNA topology and gene regulation [1,2,3]. Bacteria encode a number of NAPs that perform different functions in DNA organization and gene regulation through DNA bending, stiffening, bridging, bunching and wrapping [4]. At least twelve NAPs have been identified in *E. coli* [5], including HU, IHF, H-NS and Fis [4]. The HU protein is one of the most abundant DNA-binding proteins in *E. coli* and plays a role in organization of the bacterial chromosome [4]. Although it is referred to as a histone-like protein, HU shows no similarities at either the sequence or the structure level to eukaryotic histones [6]. Indeed, close functional relatives of HU in eukaryotes are probably high-mobility group (HMG) proteins, which are capable of bending DNA [6,7,8]. Biochemical and electron microscopic studies have revealed that HU bends and coils DNA [9,10,11] and introduces negative supercoils into covalently closed DNA in the presence of topoisomerase I activity [12]. Crosslinked HU/DNA complexes have the appearance of a beaded nucleosome-like structure under electron microscope [13], although such features were not observed without crosslinking [14,15]. HU binds to DNA with little sequence specificity, although it appears to prefer binding sites associated with kinked DNA, four-way junctions [16,17], cruciforms [18,19], DNA loops [20], single-stranded nicks or gaps [21] and nucleoprotein structures such as type I transpososomes of bacteriophage Mu [22]. High-affinity structure-specific binding is required for HU to function in specific processes, such as site-specific recombination, DNA repair, DNA replication initiation and gene regulation [23,24,25]. However, the estimated number of HU molecules (~30,000 per cell) appears far greater than these specific binding sites in cells [26], indicating that low-affinity general binding is involved in DNA condensation. Further, chromatin–immunoprecipitation coupled with DNA sequencing (ChIP-Seq) revealed that HU adopted a uniform binding mode across the genome, suggesting the non-sequence-specific binding of the protein in *E. coli* [27]. Although no stable nucleosome-like HU/DNA structures have so far been isolated from bacteria, the nucleoid was “decondensed” in *E. coli* strains lacking HU, confirming the role of the protein in DNA compaction [28]. Assuming all HU is bound to DNA, it would cover only 20% of the entire *E. coli* chromosome in an exponentially growing cell, producing a stoichiometry of one HU dimer for every ~150 bp of the DNA. The relatively low cellular abundance of HU in *E. coli* suggests that the genomic distribution of the protein is modulated in vivo. Since the formation of chromatin filaments requires high-density binding of DNA by HU (i.e., one HU dimer per 9–20 bp DNA) [15,29], flexible bends more likely occur in genomic DNA coated by HU, which could cause a reduction in the persistence length of DNA, as shown by magnetic tweezer experiments in vitro [15]. In addition, DNA networking or bunching mediated by the HU–HU multimerization triggered by sequence-non-specific DNA binding may contribute substantially to DNA condensation by bringing distant or nearby chromosome segments together [29].

The observation that HU constrains negative DNA supercoils [30,31,32] suggests that HU–DNA interactions may be influenced by the superhelical density of DNA. In other words, binding of HU to DNA may be modulated by the variation in supercoiling. It is known that chromosomal DNA is negatively supercoiled in *E. coli*. The level of supercoiling is controlled by the activities of topoisomerases. Based on several measurements, the superhelical density of DNA is in the range of −0.05 to −0.08, with an average of −0.06 in *E. coli* [33]. In addition, DNA supercoiling is neither constant nor uniform in the cell [34,35,36,37]. According to the twin-supercoiled domain model, a DNA sequence, when transcribed, experiences local and temporal changes in supercoiling—positive supercoil waves in front of and negative supercoil waves behind the moving transcription ensemble [36]. Thus, domains of highly negative, negligible or even positive supercoiling may exist transiently and locally in the cell [38,39]. In addition, cellular energetics and environmental factors, such as anaerobiosis and osmolarity, have been shown to influence the superhelical density of DNA [34,35,37]. Therefore, it is tempting to speculate that the distribution of HU in a nucleoid may respond dynamically to the fluctuation in DNA supercoiling.

Although the ability of HU to constrain negative supercoils implies that binding of HU to negatively supercoiled DNA is thermodynamically preferred, conflicting results concerning the affinity of HU for different topological forms of DNA remain to be clarified [12,40,41]. Furthermore, a quantitative description of the relationship between DNA supercoiling and HU binding is lacking. In this study, using tritium-labeled HU protein, we investigate the binding of HU to plasmid DNA with varying superhelical densities and the change in the affinity of HU for negatively supercoiled DNA as a function of the HU/DNA ratio. We find that the affinity of HU for negatively supercoiled DNA increases with increasing negative superhelical density within a range of physiological relevance. Using a chemical crosslinker, we show that the protein–protein interactions between HU dimers bound to negatively supercoiled DNA differ from those on relaxed DNA. Our results suggest that HU is disposed in response to the change in DNA supercoiling in the cell.

## 2. Materials and Methods

### 2.1. Overproduction and Purification of HU Protein

To overproduce the two HU subunits simultaneously, the *hup*B and *hup*A genes were cloned in tandem into plasmid pRLM76 and placed under the control of the λ pL promoter [42]. Plasmid containing the desired insert in a correct orientation is designated pRLM118. It was introduced into *E. coli* strain BE257*rec*A (C600 *leu*, *pro*, *lac*, *ton*A, *str*, *rec*A) to generate strain RLM1078.

For the overproduction and purification of recombinant HU, strain RLM1078 was grown in LB broth containing 50 μg/mL ampicillin at 30 °C with vigorous aeration to an OD_595_ of 2. Thermal induction was carried out either by transferring the culture to a 42 °C shaking water bath or by mixing the growing culture with 2/3 volume of pre-warmed medium (64 °C). Incubation was continued for 40 to 60 min at 42 °C. Cells were harvested (5000× *g*, 10 min, 4 °C), washed once with cold buffer A (25 mM Hepes-KOH, pH 7.6, 1 mM DTT, 0.1 mM EDTA), and resuspended in 1–2% of the original volume of the same buffer. Stock solutions of KCl and egg white lysozyme were added to the cell suspension to final concentrations of 1 M and 0.25 mg/mL, respectively. Following incubation for 30 min on ice, the sample was frozen in liquid nitrogen. The frozen sample was thawed in stirred water at 10 °C. This freeze–thaw step was repeated once. The lysate was cleared by centrifugation (100,000× *g*, 1 h, 4 °C). The supernatant was dialyzed overnight against 100 volumes of buffer B (buffer A + 10% glycerol) at 4 °C. The dialyzed material was loaded onto an S Sepharose (fast flow) column (30 mL, GE Healthcare), which had been equilibrated with buffer B. The column was washed with buffer B (30 mL) and developed with a NaCl gradient (300 mL) from 0 to 0.6 M in buffer B. HU was eluted at approximately 0.4 M NaCl. Peak fractions were pooled and concentrated on a PM-10 membrane in an Amicon ultrafiltration unit. The sample was repeatedly diluted with buffer C (50 mM Tris-Cl, pH 7.5, 1 mM DTT, 1 mM EDTA, 10% glycerol) containing 50 mM NaCl and reconcentrated to bring down the NaCl concentration in the sample to approximately 50 mM. The resulting sample was loaded onto a double-stranded DNA cellulose column (35 mL, ~1 mg DNA per ml of packed resin) that had been equilibrated with buffer C + 50 mM NaCl. The column was step-eluted with buffer C containing 50, 150 and 400 mM NaCl. HU came off the column in buffer C + 400 mM NaCl. HU from the DNA–cellulose column was electrophoretically pure but occasionally contained trace amounts of nuclease. To remove nuclease, HU was concentrated and loaded in buffer C onto a Heparin-agarose column (5 mL, Sigma) equilibrated with buffer C. The column was step-eluted with buffer C containing 0.05, 0.45 and 0.6 M NaCl. HU in the flow-through and 0.6 M wash was concentrated. Pure HU was stored in 50 mM Tris. Cl, pH 7.5, 1 mM DTT and 20% glycerol at −70 °C. All the column chromatography steps were carried out at 4 °C.

HU proteins were monitored by SDS-polyacrylamide gel electrophoresis. The nuclease contamination of HU was tested by incubating the protein with supercoiled plasmid DNA for 12 h at 37 °C in 10 mM Tris. Cl, pH 7.9, 10 mM MgCl_2_, 50 mM NaCl, 1 mM DTT and 100 μg/mL bovine serum albumin (BSA) and by running the reaction mixtures on an agarose gel. The concentration of purified HU was determined by trichloroacetic acid-Lowry assays, using bovine serum albumin as the standard. We found that the HU concentration determined by this method was higher than that by amino acid analysis by a factor of 1.18. Thus, we routinely multiplied the Lowry results by 1.18 to obtain HU concentrations.

### 2.2. Tritium Labeling of HU Protein

For labeling of recombinant HU, RLM1078 was grown to an OD_595_ of 2 at 30 °C with shaking in 300 mL of M9 minimal medium supplemented with 0.1 mg/mL thiamine, 40 μg/mL leucine, 150 μg/mL proline, 50 μg/mL each of the remaining 18 amino acids and 50 μg/mL ampicillin. Cells were harvested and resuspended in 180 mL of the supplemented M9 medium lacking leucine. Incubation was continued for 40 min at 30 °C to starve cells for leucine. The culture was then rapidly mixed with 120 mL of the supplemented M9 medium that contained 20 mCi of [^3^H]leucine (ICN) and 800 μg of unlabeled leucine and had been equilibrated to 64 °C. Following incubation for 50 min at 42 °C, cells were harvested and lysed as described above. The labeled HU was purified to apparent chemical and radiochemical homogeneity using the procedure described above. The specific activity of the purified [^3^H]HU was 6.3 × 10^5^ cpm/μg or 11,800 cpm/pmol dimer, assuming that HU exists as heterodimers.

### 2.3. Topoisomers of Plasmid pRLM4

Plasmid pRLM4, a λ*dv*-like plasmid that contains the replication origin of bacteriophage λ, has been described [43]. Native pRLM4 was purified by the alkaline lysis procedure [44] followed by two cycles of CsCl gradient centrifugation in the presence of ethidium bromide. Negatively supercoiled plasmid pRLM4 preparations differing in average linking number were prepared as follows. Native pRLM4 (0.1 mg/mL) was incubated with topoisomerase I (wheat germ) in 50 mM Tris-Cl, pH 7.5, 50 mM KCl, 10 mM MgCl_2_, 0.1 mM EDTA, 0.5 mM DTT, 30 μg/mL bovine serum albumin (BSA) and 12–100 μM ethidium bromide for 3 h at 37 °C in the dark. SDS and EDTA (both at 37 °C) were then added to the reaction mixtures to 0.2% and 20 mM, respectively. Samples were extracted with phenol and precipitated with ethanol. Relaxed, covalently closed circular pRLM4 was made in the same way, except that ethidium bromide was omitted from the relaxation reaction. Positively supercoiled pRLM4 was made as described [45], except that nicked pRLM4 was incubated with DNA gyrase for 15 min, followed by ligation for 90 min at room temperature (23 °C). The average linking differences of the above plasmid samples, except for the most negatively supercoiled and positively supercoiled ones, were determined by resolving topoisomers in the samples in agarose gels (1.2%) in the presence of proper concentrations of chloroquine diphosphate in 0.5 × TPE buffer [44] and subsequent band counting [46]. The average linking differences of the most negatively supercoiled and positively supercoiled DNA samples were extrapolated from the results of the above measurements, assuming linearity. Nicked pRLM4 was prepared as described [45].

Preparations of native pRLM4 and its topological derivatives usually contained a small fraction of catenated forms. The monomeric form of pRLM4, which was used in competition assays, was isolated by agarose gel electrophoresis and subsequent electroelution and purified by phenol extraction and ethanol precipitation.

### 2.4. Gel Retardation Assays

[^3^H]HU or unlabeled HU was mixed with plasmid pRLM4 in a binding buffer containing 20 mM Tris. Cl, pH 7.6, 1 mM DTT, 0.1 mM EDTA, 50 mM KCl, 50 mg/mL BSA and 10% (*w*/*v*) glycerol in a final volume of 20 μL. After incubation for 10 min at room temperature, the samples were subjected to electrophoresis in agarose gel (0.8%) at 4 V/cm for 3.5 h at room temperature in 25 mM Tris-glycine and 1 mM EDTA (0.5 × TGE). The gel was stained with ethidium bromide. To quantify the amounts of [^3^H]HU in HU/DNA complexes, ethidium bromide-stained DNA bands were located on an ultraviolet transluminator and excised from the gel. The gel slices were solubilized in 21% H_2_O_2_ and 17% HClO_4_ in tightly capped glass scintillation vials. Here, 15 mL of Aquasol scintillation fluid (New England Nuclear Corp., North Billerica, MA, USA) was added to each vial. Radioactivity was counted in the ^3^H channel of a Beckman liquid scintillation counter. Counting efficiency, as determined by using gel slices containing known amounts of [^3^H]HU, was close to 100%.

The relative affinity of HU for different forms of pRLM4 was determined in competition assays. A 2.2-kb double-stranded linear DNA fragment, generated by double digestion of pRLM4 with EcoRI and PstI endonucleases, was used as the reference DNA. Tested competitors included linearized, relaxed and supercoiled pRLM4. A competitor was mixed with the reference DNA in the binding buffer. [^3^H]HU was then added to the mixture. After incubation, complexes of [^3^H]HU with the reference and competitor DNA samples were separated by electrophoresis, and the radioactivity associated with each complex was determined as described above.

### 2.5. Topological Assays

Native pRLM4 (0.8 μg) was incubated with either gyrase or wheat germ topoisomerase I in the presence of HU (0–1.2 μg) for 30 min at 30 °C in 40 mM Hepes-KOH, pH 7.6, 11 mM MgCl_2_, 50 mM KCl, 2 mM ATP (omitted in the topo I reactions) and 100 μg/mL BSA. SDS and EDTA were added to final concentrations of 0.2% and 20 mM, respectively. Topoisomers in each sample were resolved by electrophoresis in 1.2% agarose in the presence (gyrase-treated samples) or absence (topo I-treated samples) of chloroquine.

### 2.6. Nick Closure Assay

The assay was conducted as described above for the preparation of positively supercoiled pRLM4, except that HU replaced DNA gyrase in the reaction. Briefly, [^3^H]HU (−16.3 μM) was incubated with nicked pRLM4 (5 ng) for 15 min at room temperature in 50 mM Tris. Cl, pH 7.8, 10 mM MgCl_2_, 10 mM DTT, 50 mM KCl, 26 μM NAD^+^ and 25 μg/mL BSA in a total volume of 50 μL. An aliquot (10 μL) of each sample was electrophoresed on a 0.8% agarose gel for the determination of the amount of DNA-bound HU. *E. coli* DNA ligase (16 units) was added to the remaining mixture. Following incubation for 90 min at room temperature, the reaction was terminated by the addition of SDS (0.2%) and EDTA (20 mM). The samples were run in 1.2% agarose in the presence and absence of chloroquine diphosphate (0.5 μg/mL), and the average linking difference of each plasmid was determined as described above.

### 2.7. Chemical Crosslinking

[^3^H]HU (0.5 μg, in 20 mM Hepes. KOH, pH 7.6, 20% glycerol) and pRLM4 (0.05–5 μg, in 10 mM Hepes. KOH, pH 7.6, 0.1 mM EDTA) were combined in 20 mM Hepes. KOH, pH 7.6, 50 mM KCl and 0.06 mM EDTA in a final volume of 25 μL. Binding reactions were for 10 min at room temperature. A solution (5 μL) of 60 mM 1-ethyl-3 (3-dimethylaminopropyl) carbodiimide (EDC, Pierce) and 30 mM N-hydroxylsulfosuccinimide (Sulfo-NHS, Pierce) was added. Following incubation for 2 h at room temperature, reactions were stopped by the addition of the sample buffer for SDS-polyacrylamide gel electrophoresis. Samples were resolved by SDS-PAGE (5–20% acrylamide). Following electrophoresis, the gel was gently rocked in 25 (*v*/*v*) methanol and 10% (*v*/*v*) acetic acid for at least 1 h, in En^3^Hance (Du Pond) for 1 h and in water for 30 min. The gel was then dried and exposed to X-ray film at −80 °C. The effect of the HU concentration on the crosslinking of HU in the absence of DNA was examined by mixing [^3^H]HU (0.5 μg) with increasing amounts of unlabeled HU (0–23.1 μg) in a total volume of 20 μL. The mixtures were crosslinked and the crosslinking products were analyzed as described above.

## 3. Results

### 3.1. Binding of HU to Negatively Supercoiled and Nicked Circular DNA

**Figure 1:**
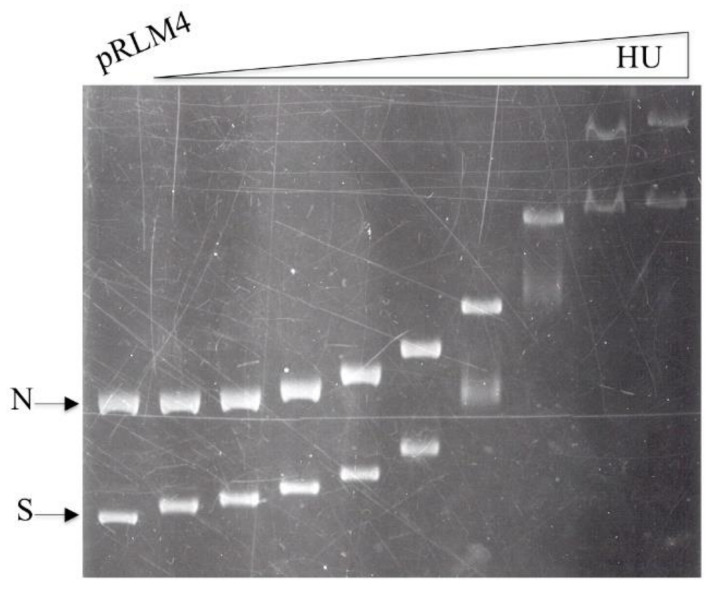
Gel retardation of negatively supercoiled and nicked DNA by HU. Negatively supercoiled (S) and nicked (N) pRLM4, each at 100 ng, were incubated with increasing amounts of HU (0, 10, 20, 50, 100, 200, 400, 800, 2000 and 4000 ng) in 25 μL for 10 min at 23 °C. Aliquots (20 μL) of the mixtures were electrophoresed on a 0.8% agarose gel.

A mixture of negatively supercoiled and nicked pRLM4 at a mass ratio of one was titrated with HU and the resultant HU/DNA complexes were resolved by electrophoresis in agarose. Similar retardation patterns were obtained for the two forms of DNA (Figure 1). These retardation patterns were not affected by the following modifications in assay conditions: omission of glycerol from or addition of Mg^2+^ ions (12 mM) to the binding mixture; electrophoresis at 4 °C for a longer time (6 h) or in 0.6% agarose; no incubation of the binding mixture. Obviously, binding of HU to DNA rapidly reached an equilibrium, and once formed HU/DNA complexes were stable under the tested electrophoretic conditions.

Despite their similarity, the two binding patterns are appreciably different at low HU/DNA ratios. The supercoiled plasmid appears to be retarded preferentially at low HU concentrations, suggesting that HU may have higher affinity for negatively supercoiled DNA than for nicked DNA.

**Figure 2:**
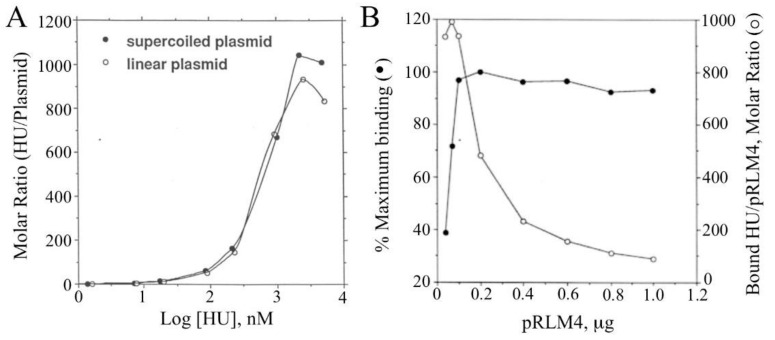
Binding of HU to negatively supercoiled and linear DNA. (**A**) [^3^H]HU was titrated into a binding mixture containing 0.2 μg of negatively supercoiled or linear pRLM4. After incubation for 10 min at 23 °C, [^3^H]HU–DNA complexes were resolved by electrophoresis on a 0.8% agarose gel. The gel was stained with ethidium bromide, retarded DNA bands were visualized on a UV transluminator and excised from the gel. DNA-bound HU was quantitated by measuring the radioactivity in the gel slices as described in Section 2. Molar ratios of bound HU dimers to pRLM4 were calculated. (**B**) [^3^H]HU (1.27 μM) was incubated with increasing amounts of linear pRLM4. [^3^H]HU–DNA complexes were processed as described above.

Taking advantage of the use of tritium-labeled HU in the assay, we determined the amount of HU associated with each retarded DNA band or in each HU/DNA complex. Control experiments showed that in a typical agarose gel, smearing free [^3^H]HU accounted for 2–5% of the radioactivity of an HU/DNA band, and this amount was subtracted. Two HU binding curves are shown in Figure 2A. Both negatively supercoiled and linear plasmid DNA samples appeared to be saturated by similar amounts of HU, i.e., ~1000 HU dimers per pRLM4 molecule. In other words, one HU dimer bound five to six base pairs of DNA, since pRLM4 is 6148 bp long. Analysis of the relationship between the extent of retardation and the molar ratio of bound HU dimer to pRLM4 revealed that the largest decrease in mobility of an HU/pRLM4 complex occurred when DNA was about 50% saturated with HU or when one pRLM4 molecule was bound by 500 HU dimers. It is known that the rate of migration of a protein/DNA complex in a gel is the function of the mass, net charge and conformation of the complex [47]. The observed transition in the retardation pattern is probably due to the conformational change of the HU/DNA complex, since no significant change in binding equilibrium was detected across the point of transition. Whether the plasmid was negatively supercoiled or nicked, the transition appeared to take place at the same HU/DNA ratio.

Similar results were obtained when HU was incubated with various amounts of linear pRLM4 DNA (Figure 2B). At 1.27 μM HU, the amount of HU bound to the DNA was constant and independent of the molar ratio of bound HU to DNA under subsaturation binding conditions. This titration again indicates that the binding capacity of pRLM4 for HU is approximately 1000 dimers per DNA molecule.

### 3.2. Affinities of HU for Different Forms of pRLM4

**Figure 3:**
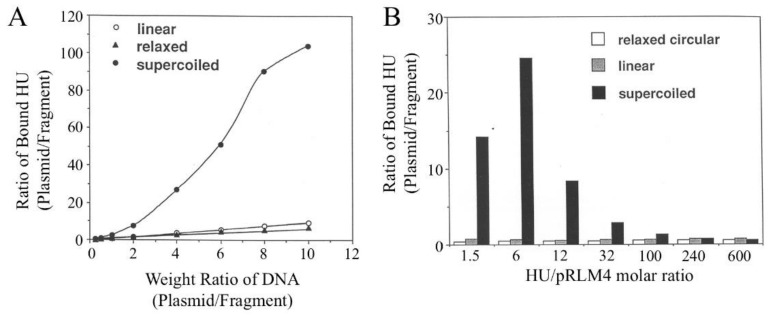
Competition for HU binding between a linear DNA fragment and various forms of pRLM4. Competition assays contained [^3^H]HU, a 2.2-kb linear DNA fragment (the reference) and one of the following competitors: negatively supercoiled, relaxed or linear pRLM4. After incubation for 10 min at 23 °C, the binding mixtures were electrophoresed on agarose gels. HU bound to the reference DNA and to the competitor were determined as described in Section 2. The binding ratio represents the ratio of HU bound to a competitor DNA to that bound to the 2.2-kb fragment. In (**A**), the levels of HU (140 nM) and the 2.2-kb fragment (0.1 μg) were constant, while the amount of each competitor was varied. In (**B**), the levels of the 2.2-kb fragment (0.1 μg) and each competitor (0.1 μg) were constant, whereas the amount of HU was varied.

To compare quantitatively the affinities of the HU protein for negatively supercoiled, relaxed and linear DNA samples, we employed a binding competition assay. In the assay, different forms of plasmid DNA were allowed to compete with a 2.2-kb EcoRI-PstI fragment of pRLM4 (the reference) for binding by HU under subsaturating conditions. Binding of HU to the reference and to a competitor were compared at various competitor/reference DNA ratios (Figure 3A) or HU concentrations (Figure 3B). As shown in Figure 3A, the partition of HU between the 2.2-kb fragment and the relaxed or linear plasmid was proportional to the mass ratio of the two competing DNAs, suggesting that HU has similar affinities for relaxed and linear DNAs. In contrast, binding of HU to supercoiled plasmid was clearly favored over that to the reference DNA, and this binding preference of HU was more pronounced at higher plasmid/fragment ratios. The affinity of HU for negatively supercoiled DNA as a function of the molar ratio of bound HU to plasmid was further examined in a wide range of HU concentrations (Figure 3B). It seemed that HU bound most strongly to the negatively supercoiled plasmid at an HU/pRLM4 molar ratio of six, a number corresponding to ~0.6% of the maximum binding capacity of the plasmid for HU. The affinity of HU for the supercoiled DNA was about 20 times greater than that for linear DNA at this ratio. The preference of HU for negatively supercoiled DNA diminished when the plasmid was bound by more than 100 HU dimers or saturated by 10%. However, no reversal of the binding preference was found even at the highest HU/pRLM4 ratio. When the HU/pRLM4 became smaller than six, the difference in affinity of HU for the two forms of DNA surprisingly decreased. Based on the observation that HU tetramers were more readily crosslinked on negatively supercoiled DNA than on linear DNA (see below), we suggest that the high affinity of HU for supercoiled DNA may result from supercoiling-dependent or supercoiling-enhanced protein–protein interactions between bound HU dimers. At very low HU/DNA ratios, these interactions may become infrequent, meaning the binding affinity decreases. Similar relationships between the affinity of HU for negatively supercoiled DNA and the ratio of bound HU to DNA were found when two other plasmids, pUC19 and pRLM76, instead of pRLM4 were used. From these experiments, we conclude that the HU protein preferentially binds to negatively supercoiled DNA under subsaturating conditions and that the binding affinity depends strongly on the HU/plasmid molar ratio.

Upon binding to covalently closed circular DNA, HU is capable of constraining negative DNA supercoils, thereby relaxing regions of DNA that are not bound by HU. Presumably, the HU/plasmid-ratio-dependent change in binding affinity of HU for negatively supercoiled DNA correlates with the topological consequence of HU binding to the DNA.

### 3.3. Binding of HU to pRLM4 Topoisomers

**Figure 4:**
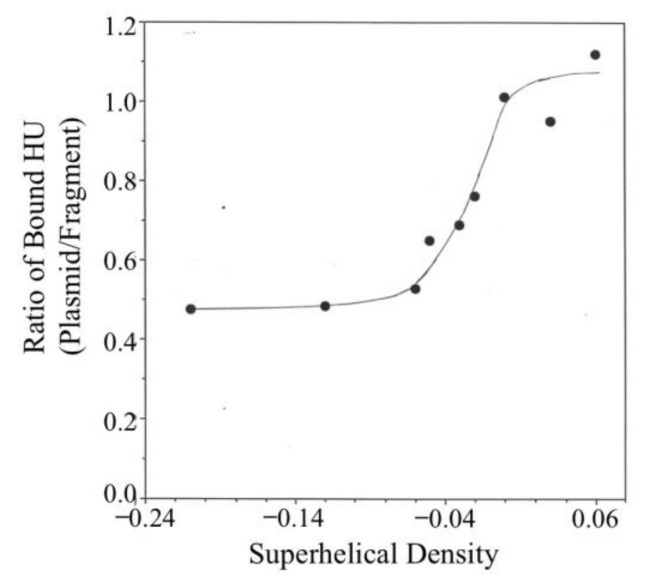
Effect of the superhelical density of DNA on HU binding. The assays were conducted as described in Figure 3, except that competitors were pRLM4 preparations differing in average linking number. R (1/2) represents the mass ratio of a competitor to the 2.2-kb fragment at which binding of HU to the fragment was 50% of that in the absence of the competitor.

Native plasmid pRLM4 isolated from its host *E. coli* strain contains approximately 35 negative supercoils and has a superhelical density of –0.06. It has been estimated that less than half of the linking deficit exists in a free, accessible state in vivo [11,48,49,50]. In addition, global and local changes in DNA supercoiling are believed to take place constantly. How does DNA supercoiling affect binding of HU to DNA? To answer this question, we expanded the binding competition assay to include pRLM4 with varying superhelical densities as competitors. These competitors were compared for their ability to reduce binding of HU to the 2.2-kb fragment. Competitor/reference weight ratios, at which the amount of HU bound to the fragment was 50% of that found in the absence of a competitor plasmid, are shown in Figure 4. It seemed that binding of HU to DNA was significantly affected by a change in superhelical density only within a narrow but physiologically relevant range (i.e., –0.06 to 0). The affinity of HU increased only slightly when the superhelical density of the plasmid was reduced from –0.06 to –0.10. On the other hand, the affinity of HU for positively supercoiled DNA was marginally lower than that for relaxed DNA.

The data in Figure 4 appear to suggest a rather moderate effect of the variation in the degree of DNA supercoiling on the binding affinity of HU for DNA. This is because the amounts of HU bound to the supercoiled competitor plasmids at the competitor/reference ratios needed to achieve the desired reduction of binding of HU to the reference fragment were relatively large. For instance, the amounts of HU bound to the two most negatively supercoiled competitors (*σ* = −0.06 and −0.10) were approximately 6–9% of the saturating level. As shown in Figure 4, at this level of binding, the difference between the affinity of HU for the negatively supercoiled plasmid (*σ* = −0.06) and that for the relaxed plasmid is merely two-fold. At lower HU/competitor ratios, the binding affinity of HU to DNA became more sensitive to the changes in DNA supercoiling.

### 3.4. Constraining of Negative Supercoils by HU

**Figure 5:**
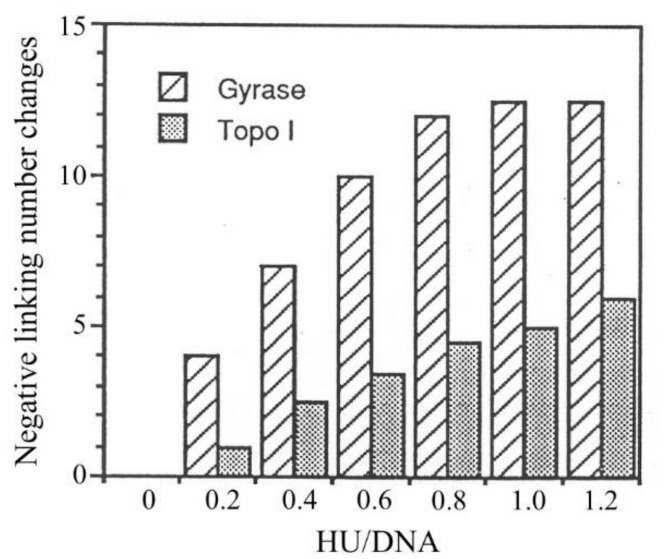
Effect of HU on topoisomerase-mediated linking change of pRLM4 under equilibrium conditions. Native pRLM4 (0.8 μg) was incubated with either gyrase or eukaryotic topoisomerase I in the presence of HU (0–1.2 μg) for 30 min at 30 °C. Reactions were stopped by the addition of SDS and EDTA. The topoisomers in each sample were resolved by electrophoresis in 1.2% agarose in the presence (gyrase-treated samples) or the absence (topo I-treated samples) of chloroquine. The linking change was determined by band counting. In the absence of HU, the plasmids contained 0 and 33 negative supercoils, respectively, following the treatment with topo I and gyrase.

The affinity of HU for negatively supercoiled DNA depends on the molar ratio of bound HU to DNA. As more HU binds to the DNA, the binding affinity decreases. In view of the ability of HU to constrain negative supercoils, we sought to determine if the dependence of the binding preference of HU for negatively supercoiled DNA on the HU/DNA ratio correlates with the change in the superhelical tension of the template as a result of HU binding. The number of negative supercoils taken up by HU has traditionally been determined in DNA relaxation assays involving the use of eukaryotic topoisomerase I. The unbound DNA is relaxed at equilibrium in these assays. Therefore, it was of interest to determine the ability of HU to constrain supercoils on negatively supercoiled DNA. In the present study, we compared the linking changes of a plasmid in topo I-mediated relaxation and gyrase-mediated supercoiling reactions as a function of the HU/DNA ratio (Figure 5). Assuming that the two topoisomerases act only on HU-free DNA, and either relax or supertwist it to a fixed level, the linking change should reflect the number of supercoils constrained by the protein. In the gyrase reaction, pRLM4 had 33 negative supercoils (*σ* = −0.06) at equilibrium whether the plasmid was originally relaxed or natively supercoiled. It is clear from Figure 5 that the linking change of the plasmid mediated by either topo I or gyrase was affected by HU. The linking change of pRLM4 in the gyrase reaction was significantly greater than that in the topo I reaction at the same HU/DNA ratios. The number of supercoils constrained by HU in the topo I assay appeared to be proportional to the HU/DNA ratio within the tested range. However, the HU-mediated linking change in the gyrase assay did not increase linearly with the HU/DNA ratio, presumably because the action of gyrase was affected at high HU/DNA ratios. Consequently, the HU-mediated linking changes in the two assays differed by 4-fold at an HU/DNA ratio of 0.2 and by 2.5-fold at an HU/DNA ratio of 1. Since the equilibrium states of supercoiling in the protein-free regions of DNA are different in the two reactions, it is tempting to suggest that HU constrains more negative supercoils on negatively supercoiled DNA than on relaxed DNA. However, at present, we cannot rule out other explanations. For example, HU may directly affect the supercoiling activity of gyrase. However, even if this is the case, the effect is probably insufficient to account for the observed difference between the HU-mediated linking change in the gyrase reaction and that in the topo I reaction. It is worth noting that at the concentrations used in the experiment, HU molecules should all bind to DNA regardless of the topological state of the DNA. Therefore, the difference in the capacity of HU to constrain supercoils, as observed in the two topological assays, is not due to the difference in HU binding. This is consistent with the notion that binding of HU to DNA does not necessarily result in constraining negative supercoils [51,52].

**Figure 6:**
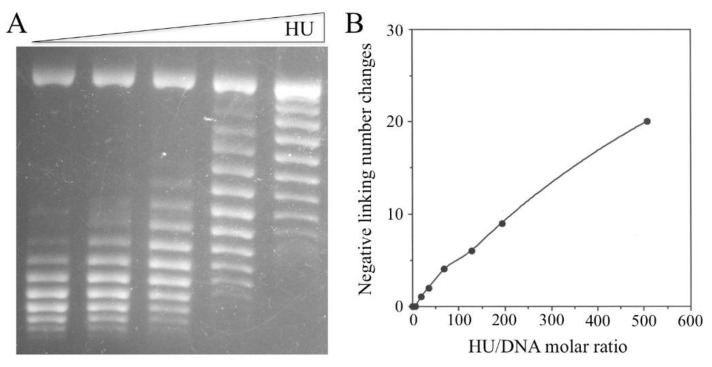
Linking change of circular plasmid DNA covalently closed in the presence of HU. Plasmid pRLM4 (1 μg) containing a single nick was incubated with various amounts of [^3^H]HU (0, 0.225, 0.45, 0.675, 0.9, 1.35, 1.8 and 3.6 μg) in 50 μL for 15 min at 23 °C, and an aliquot (40 μL) of each sample was subsequently ligated with *E. coli* DNA ligase (16 U) for 1.5 h at 23 °C. Ligation reactions were terminated by the addition of SDS and EDTA. The amount of bound [^3^H]HU was determined by running an aliquot of the pre-ligation mixture on agarose gel and quantitating [^3^H]HU in the [^3^H]HU–DNA complex as described in Section 2. The linking change of DNA was measured by resolving topoisomers on agarose gel in the presence and absence of chloroquine and band counting. (**A**) Agarose gel (0.5% chloroquine) electrophoresis of plasmid pRLM4 ligated in the presence of HU (0, 0.225, 0.45, 0.675 and 0.9 μg). (**B**) Linking change of the ligated pRLM4 as a function of bound HU dimers.

The number of HU dimers required to constrain one negative supercoil, as determined in the topo I assay in this study, was much higher than previously reported estimates. In order to determine accurately the stoichiometric relationship between the HU/DNA ratio and the number of constrained supercoils, we performed the following two assays in parallel using [^3^H]HU: a nick closure assay to measure the number of supercoils constrained by HU and a gel shift assay to quantify DNA-bound HU. The use of either *E. coli* DNA ligase or bacteriophage T4 DNA ligase produced the same results. As shown in Figure 6, the number of constrained supercoils increased linearly with the amount of bound HU when the molar ratio of HU dimer to pRLM4 ranged from 0 to 500 or the HU/pRLM4 mass ratio ranged from 0 to at least 2.2:1. The constrained supercoils were negative, as evidenced by the effect of chloroquine, an intercalator that reduces the rotational angle in DNA double helixes on the mobilities of the topoisomers of the ligated pRLM4 in gels. The ligation products could not be resolved into topoisomers and acquired unexpected mobilities at HU/DNA mass ratios greater than 2.2:1. The ligation efficiency, as estimated on the basis of the amount of residual unligated DNA in each reaction, was not markedly affected by the amount of bound HU, except at HU/DNA ratios where ligation products became unresolvable under our conditions. Since the plasmid to which HU bound was relaxed, the data indicate that HU protein is capable of constraining negative supercoils in the absence of energetically favorable torsional tension.

To determine the rate at which DNA is wrapped around HU, we incubated nicked plasmid pRLM4 with HU for various lengths of time and ligated the DNA for 30 s. We found that the number of negative supercoils trapped in the ligated plasmid at the first time point (30 s) was the same as that at the last time point (90 min). Taking the ligation time into consideration, wrapping of DNA around HU was complete within 1 min of the addition of HU to the DNA.

The ability of HU to take up negative supercoils seemed to be rather limited, since approximately 20 bound HU dimers were needed for the linking change of one supercoil (Figure 6B). This estimate of the stoichiometry differs significantly from those reported previously. In an early study, Rouviere-Yaniv et al. [13] observed bead-like features of the complexes between HU and SV40 DNA fixed with glutaraldehyde under electron microscope. Later, Broyles and Pettijohn [12] obtained a similar stoichiometry of 19 HU monomers for each supercoil in a topoisomerase-based assay. They also found that DNA became maximally supercoiled at an HU/DNA weight ratio of 1 and less supercoiled at higher ratios. The discrepancy between our data and the previous results most likely arises from the difference in the way the amount of bound HU is determined. In both of the previous studies, the amount of bound HU was assumed to be similar to the total amount of HU used in the reaction. This assumption appears to hold, since most of the input HU is bound to DNA at the concentrations used in those studies. However, the amount of HU in each binding reaction may have been incorrectly assumed. For example, the dye-binding method of Bradford, used by Broyles and Pettijohn, has been found to seriously underestimate HU concentration [53] (Huang and McMacken, unpublished observation). Added to the complexity of the problem is the tendency of HU to bind to the surface of various containers, such as microcentrifuge tubes and test tubes. In this study, the use of [^3^H]HU, whose specific radioactivity was calculated on the basis of the adjusted Lowry concentration of the protein, permitted a direct measurement of the amount of HU in an HU–DNA complex resolved by gel electrophoresis. The difference in the measurement of HU concentration may also explain why we observed a linear increase in the number of supercoils with increasing amounts of bound HU over a wider range of HU/DNA mass ratios than previously reported [12]. Interestingly, the unresolvable nick closure products started to appear at HU/DNA ratios where the biggest drop in the mobility of an HU/DNA complex in the gel retardation assay took place (Figure 1). Therefore, it seems that DNA, when bound by HU to 50% saturation, undergoes a drastic conformational change.

### 3.5. Chemical Crosslinking of DNA-Bound HU

**Figure 7:**
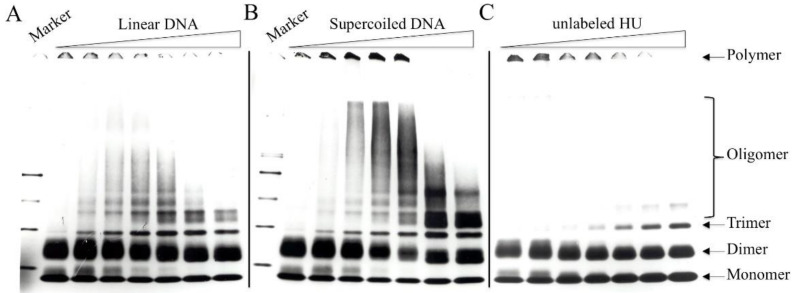
Chemical crosslinking of HU protein. [^3^H]HU (0.5 μg) was incubated with linear (**A**) or negatively supercoiled (**B**) pRLM4 (0, 0.05, 0.1, 0.25, 0.5, 1.5 and 5 μg) or unlabeled HU (0, 0.5, 2, 4.5, 9.5, 14.5 and 23.1 μg) (**C**) for 10 min at 23 °C. EDC (10 mM) and Sulfo-HNS (5 mM) were added to the mixture. After 2 h at 23 °C, reactions were stopped by the addition of the sample buffer for SDS-PAGE. Crosslinked products were resolved by SDS-PAGE (5–20% acrylamide). The gel was dried and processed for fluorography as described in Section 2.

Structural studies predict protein–protein interactions between HU molecules that are bound to DNA. In this study, the protein–protein interactions of DNA-bound HU were probed with EDC, a carbodiimide zero-length crosslinker capable of introducing isopeptide bonds between carboxyl and amino groups of amino acid side chains in proteins [54]. [^3^H]HU (0.5 μg) was mixed with varying amounts of either linear or supercoiled pRLM4 (0–5 μg). The HU/DNA complexes were incubated with EDC (10 mM) for 2 h at room temperature in the presence of sulfo-HNS (5 mM), which enhanced EDC coupling. In a control experiment, BSA was not crosslinked under the same conditions. Crosslinked HU complexes were separated by gel electrophoresis under denaturing conditions. HU in solution was not efficiently crosslinked into oligomers larger than dimers with EDC (Figure 7). Dimers remained as a predominant crosslinked product when the concentration of HU was raised to 0.9 mg/mL. When bound to DNA, however, HU was readily crosslinked into high molecular weight oligomers at a concentration of 0.02 mg/mL (Figure 7C). DNA itself was not crosslinked to HU or entangled in crosslinked HU oligomers, since treatment of crosslinked samples with DNA cleaving agents did not alter the electrophoresis pattern. The size distribution of the crosslinked products depended strongly on the mass ratio of HU to DNA—larger HU oligomers were formed at higher HU/DNA ratios (e.g., 5:1) (Figure 7A,B). The reduced band intensity in the high molecular weight region at an HU/DNA mass ratio of 10:1 was presumably due to the decrease in the amount of bound HU.

Interestingly, even-numbered HU oligomers (e.g., dimers and tetramers) appeared as doublets, whereas odd-numbered HU oligomers (e.g., trimers and pentamers) were single bands. HU oligomers crosslinked in the absence of DNA had the same migration pattern. Losso et al. [55] made a similar observation in their crosslinking studies of HU using dimethylsuberimidate and dimethyladipimdate as crosslinking reagents. The presence of two migrating species for each even-numbered HU oligomer is not due to the heterotypic nature of HU, since crosslinking of either HU-1 or HU-2 alone produced a similar result. Why are there two crosslinking products for an even-numbered HU oligomer? Presumably, there exist two distinctly different sets of crosslinkable contacts between two HU monomers. We interpret the migration pattern of crosslinked HU oligomers as follows. Assuming that (A) an HU monomer possesses two independent binding surfaces or sites for binding by other HU monomers and (B) the two binding sites of an HU monomer may be separately bound by two HU monomers but cannot be both occupied by a single monomer, an HU monomer may be crosslinked to another in two alternative ways, yielding two different dimers resolvable by SDS-PAGE. Crosslinking of a third HU monomer to one of the unoccupied sites on an HU dimer leads to the formation of a trimer. Obviously, all trimers contain the same two sets of interactions between three monomers and have only one denatured form. Likewise, a tetramer or larger even-numbered oligomer may be viewed as constructed from HU dimers. Since one HU dimer interacts with another dimer of the same type only at one of its two remaining binding sites, an even-numbered HU oligomer with extendable ends may be formed from either, but not both, of the two types of HU dimers. Therefore, there are two types of ends for even-numbered oligomers, and a single type of end for odd-numbered oligomers. This interpretation is consistent with the predictions of the structural analysis of HU that the protein has hydrophobic surfaces for dimerization, as well as for dimer–dimer interaction. Taken together, these data suggest that HU may form a continuous protein thread with open ends when interacting with DNA.

Efficiency levels with which intermolecular contacts within an HU dimer and those between HU dimers were crosslinked appeared to be affected by HU/DNA mass ratios. In the absence of DNA, HU was crosslinked into two types of dimeric products that migrated in an SDS-PAGE gel as a doublet with two bands of similar intensities. As HU bound to an increasing amount of DNA, the relative abundance of the two crosslinked species in the doublet became increasingly shifted toward the fast-migrating one, regardless of whether supercoiled or linear DNA was used. It appears that one of the two modes of HU–HU interaction, probably that required for the dimerization of HU, is stabilized by DNA.

Crosslinking of DNA-bound HU was significantly affected by the topology of DNA. Substantially more and larger HU oligomers were crosslinked on negatively supercoiled DNA than on linear DNA. At HU/DNA mass ratios of 10:1 and 5:1, where preferential binding of HU for negatively supercoiled DNA was observed, HU tetramers were clearly a major product on supercoiled pRLM4. In contrast, far fewer HU tetramers were found on linear pRLM4 at similar HU/DNA ratios. Surprisingly, the tetrameric form of HU was detectable even when the HU/DNA ratio was reduced to an average of four HU monomers per molecule of supercoiled pRLM4. It appears, therefore, that the interactions between DNA-bound HU dimers are highly favored on negatively supercoiled DNA, and given its intracellular abundance, HU may exist predominantly as tetramers in the cell.

## 4. Discussion

The chromosomal DNA in *E. coli* is negatively supercoiled, although the degree of supercoiling is neither constant nor uniform. Transient local changes in supercoiling may occur as a result of active DNA transactions such as transcription. Topologically independent DNA loops in the chromosome may be supertwisted to various levels. We wished to understand how DNA supercoiling affects binding of the HU protein to DNA and how the topological heterogeneity of chromosomal DNA may influence the in vivo distribution of HU. In this study, we have demonstrated that HU binds more tightly to negatively supercoiled DNA than to relaxed DNA. This binding preference is more pronounced at lower mass ratios of bound HU to DNA (i.e., 1:10 and 1:5) and negligible at HU/DNA mass ratios of 1:2 and higher. Assuming two copies of chromosomal DNA per exponentially growing cell, the in vivo HU/DNA mass ratio is approximately 1:10. Because of the high cellular HU concentration (about 50 μM), stoichiometric binding conditions should exist. In other words, the mass ratio of bound HU to DNA should also be about 1:10. At this ratio, binding of HU to DNA is highly favored. Kobryn et al. [56] reported similar findings obtained by using HU chemical nucleases. We have also found that the binding affinity of HU for DNA is most sensitive to the change in supercoiling within a narrow range of superhelical density (*δ* = −0.06–0), and less so beyond this range. Depending on growth phase and environmental conditions, the unconstrained superhelical tension in chromosomal DNA fluctuates around −0.02 to −0.03, meaning it falls in the “above” range. Since HU/DNA complexes dissociate rapidly [12], the distribution of HU on chromosomal DNA is likely a dynamic process influenced by fluctuations in supercoiling. For instance, a transcribing RNA polymerase is known to create positively and negatively supercoiled domains around it [36]. However, the number of high-affinity HU binding sites generated in the negatively supercoiled domain is presumably far greater than the number of HU dimers dissociated from the positively supercoiled domain because of the low in vivo HU/binding site ratio. Therefore, actively transcribed regions of chromosome probably attract more HU than inactive regions. The significance of supercoiling-dependent binding of HU to DNA in cells remains to be understood. If HU is indeed able to move around in the chromosome in response to fluctuations in DNA supercoiling, as suggested in the present study, it may provide the cell with a mechanism for reducing deleterious or unwanted DNA activities associated with excessively underwound regions that are likely formed behind DNA-tracking enzyme complexes such as RNA polymerase. Preferential binding of HU to the underwound regions of DNA may help stabilize them.

Based on the present study, an HU dimer binds to a stretch of six base pairs of double-stranded DNA at saturation. Assuming 60,000 HU monomers per exponentially growing cell, the chromosomal DNA is only 2% saturated with HU. It is believed that about 60% of the total supercoils in genomic DNA exist in a protein-bound form in *E. coli* [48,49,50,57]. To what extent then does HU binding contribute to the constraining of negative supercoils? To address this question, we analyzed the effects of HU on DNA topology using several assays. Taking advantage of the availability of radiolabeled HU, we established the stoichiometric relationship between bound HU and constrained supercoils in a nick closure assay. The ability of HU to constrain supercoils appears to be rather insignificant, since about 20 bound HU dimers are required to take up one supercoil on relaxed DNA. Based on this number, there is only enough HU to constrain about 3% of negative supercoils or to account for 5% of the total constrained supercoils in vivo. Other NAPs, such as Fis [58] and H-NS [59], likely play an important role in constraining negative DNA supercoils. It is possible, however, that HU constrains more supercoils on negatively supercoiled DNA than on relaxed DNA, as suggested by the difference between the HU-mediated linking change in plasmid pRLM4 in the topo I assay and that in the gyrase assay. However, even when taking this possibility into account, HU does not appear to play a major role in constraining negative supercoils in the cell. This finding is consistent with the observation that the linking deficits of plasmids isolated from HU-deficient mutants were only 10–15% lower than those from wild-type cells [60,61].

It is not surprising that HU binds more tightly to negatively supercoiled DNA than to relaxed DNA, since HU is capable of constraining negative supercoils, a process which is energetically favored on negatively supercoiled DNA. The free energy of supercoiling in negatively supercoiled DNA may help stabilize HU–DNA interactions. The preferential binding of HU to negatively supercoiled DNA depends strongly on the ratio of HU to DNA and is not detectable at high HU/DNA ratios. In contrast, the binding affinity of HU for relaxed or linear DNA is independent of HU/DNA ratios. A possible explanation for the inverse relationship between the affinity of HU for negatively supercoiled DNA and the HU/DNA ratio is that upon binding, HU titrates negative supercoils, leaving unbound DNA more relaxed. However, this explanation appears to be incomplete at best for the following two reasons. First, since there are 35 negative supercoils in the plasmid used in the study, the plasmid DNA will not be completely relaxed unless an HU/plasmid molar ratio of 700:1 is reached. However, the difference between binding of HU to supercoiled DNA and that to relaxed DNA disappears at an HU/DNA ratio of 300:1. Second, the two sets of DNA topoisomers with average linking differences of −0.06 and −0.12, respectively, are similarly effective in competing against a linear DNA fragment for binding by HU. It appears, therefore, that HU binding introduces into negatively supercoiled DNA conformational changes that cannot be described solely in topological terms. Presumably, the basis for the binding preference of HU is the presence of certain types of protein–protein or protein–DNA interactions that are stable only on negatively supercoiled DNA for both energetic and structural reasons, and these interactions are sensitive to the conformational changes of supercoiled DNA brought about by HU binding.

Once bound to DNA, HU dimers are positioned to interact with one another, and the protein–protein interactions are likely the source of deformation of DNA. In order to gain an insight into these interactions, we chemically crosslinked HU bound to DNA using a zero-length crosslinker (EDC) [54] and analyzed the crosslinked products by SDS-polyacrylamide gel electrophoresis. HU is significantly more readily crosslinked on DNA, especially on negatively supercoiled DNA, than in solution. Since HU had been suggested to form nucleosome-like beads on DNA [13], we sought to determine whether an HU oligomer of a certain size would be predominant among crosslinked products. Although high molecular weight HU oligomers could not be resolved into discrete bands on the gel, the protein smears displayed no visible discontinuity, which would suggest the existence of the HU beads with sizes up to 200 kDa. The sizes of crosslinked products appear to increase with an increasing HU/DNA ratio. Interestingly, substantially more HU is crosslinked in a tetrameric form on negatively supercoiled DNA than on linear DNA at HU/DNA ratios, where the preferential binding of HU to negatively supercoiled DNA is more evident. Tetramers are formed even when there are, on average, only four HU monomers on a supercoiled plasmid. These data suggest that HU interacts with negatively supercoiled DNA primarily as a tetramer. This agrees well with the mathematical model proposed by Voulgarakis that HU-HU electrostatic interactions can force proteins to assemble in rigid spiral configurations on DNA [62]. Similar observations were made when other chemical crosslinkers were used [63,64,65]. In crystallographic studies, HU was found to be able to form dimers, although potential interactions between HU dimers were also envisioned [30,66]. We speculate that an HU dimer bound to a DNA strand is capable of dimer–dimer interactions with another HU dimer bound to a separate DNA strand, resulting in the formation of an HU tetramer that brings together two DNA strands. The protein–protein interactions involved in the formation of HU tetramers on DNA are, therefore, enhanced by DNA supercoiling. Since the association of HU tetramers with DNA is apparently energetically favorable, these interactions may contribute to the torsional tension that binding of HU brings into DNA.

## Data Availability

The data presented in this study are available upon request from L.H.
